# Design of Experiment on Concrete Mechanical Properties Prediction: A Critical Review

**DOI:** 10.3390/ma14081866

**Published:** 2021-04-09

**Authors:** Beng Wei Chong, Rokiah Othman, Ramadhansyah Putra Jaya, Mohd Rosli Mohd Hasan, Andrei Victor Sandu, Marcin Nabiałek, Bartłomiej Jeż, Paweł Pietrusiewicz, Dariusz Kwiatkowski, Przemysław Postawa, Mohd Mustafa Al Bakri Abdullah

**Affiliations:** 1 Faculty of Civil Engineering Technology, Universiti Malaysia Pahang, Kuantan 26300, Malaysia; bengwei.chong@aiesec.net; 2 Department of Civil Engineering, College of Engineering, Universiti Malaysia Pahang, Kuantan 26300, Malaysia; ramadhansyah@ump.edu.my; 3 Center of Excellence Geopolymer and Green Technology, Universiti Malaysia Perlis, Kangar 01000, Malaysia; sav@tuiasi.ro (A.V.S.); mustafa_albakri@unimap.edu.my (M.M.A.B.A.); 4 School of Civil Engineering, Universiti Sains Malaysia (Engineering Campus), Nibong Tebal 14300, Malaysia; cerosli@usm.my; 5 Faculty of Material Science and Engineering, Gheorghe Asachi Technical University of Iasi, 700050 Iasi, Romania; 6 Department of Physics, Częstochowa University of Technology, 42214 Częstochowa, Poland; nmarcell@wp.pl (M.N.); bartek199.91@o2.pl (B.J.); pawel.pietrusiewicz@pcz.pl (P.P.); 7 Faculty of Mechanical Engineering and Computer Science, Częstochowa University of Technology, 42214 Częstochowa, Poland; kwiatkowski@ipp.pcz.pl (D.K.); postawa@ipp.pcz.pl (P.P.)

**Keywords:** design of experiment, concrete properties, review, regression, response surface methodology, artificial neural network

## Abstract

Concrete mix design and the determination of concrete performance are not merely engineering studies, but also mathematical and statistical endeavors. The study of concrete mechanical properties involves a myriad of factors, including, but not limited to, the amount of each constituent material and its proportion, the type and dosage of chemical additives, and the inclusion of different waste materials. The number of factors and combinations make it difficult, or outright impossible, to formulate an expression of concrete performance through sheer experimentation. Hence, design of experiment has become a part of studies, involving concrete with material addition or replacement. This paper reviewed common design of experimental methods, implemented by past studies, which looked into the analysis of concrete performance. Several analysis methods were employed to optimize data collection and data analysis, such as analysis of variance (ANOVA), regression, Taguchi method, Response Surface Methodology, and Artificial Neural Network. It can be concluded that the use of statistical analysis is helpful for concrete material research, and all the reviewed designs of experimental methods are helpful in simplifying the work and saving time, while providing accurate prediction of concrete mechanical performance.

## 1. Introduction

Design of Experiment (DoE) is an effective tool for handling multiple variables in problem solving [1]. The method has been used to improve experimentation performance in engineering, services, and manufacturing industries [2]. Traditionally, problems with multiple variables are solved using the “one variable at a time” (OVAT) approach, which holds all but one variable constant and conducts experiments until the optimal result is obtained for the single manipulated variable. To study a problem with multiple variables, the process is repeated for each variable until the best result is achieved. While the method is simple and accurate, it still requires a large amount of samples and experiment, which consumes a lot of time, resource, and labor [3]. The problem is of highly relevance in studies involving concrete materials. While such studies focus on a single responding variable, such as the effect and amount of replacement material on concrete properties [4,5,6,7,8], the performance of high-performance and self-compacting concrete is affected by a huge number of variables that are potentially interrelated with each other [4]. Alternative concrete design with waste material also requires a large amount of studies to be implemented. In such cases, a big number of samples will be required to consider all the significant variables of concrete strength. In general, the number of combinations available for r variables which can take n numbers are denoted by the expression $n^{r}$. For example, an experiment with five variables and five values alone requires 3125 sets of data, making detailed study of concrete properties a time- and cost-consuming task that is not practical to be conducted solely through laboratory experimentation. Due to this reason, Design of Experiment (DoE) was selected, in this study, as a method to optimize the process of data collection and analysis.

DoE optimizes the number of samples required to carry out accurate statistical prediction, which explains the effect of each variable on the dependent variables being studied. However, the flexibility of DoE may present a problem, particularly for researchers without sufficient knowledge on the field, and with the absence of a clear methodology in applying the problem-solving task [5]. The application of DoE in the study of concrete materials has gained traction in recent decades. The applications of DoE in concrete studies range from the prediction of concrete strength to the development of concrete mix design. The assessment of concrete strength through non-destructive tests often requires mathematical formulation, as it is not possible to deduce the strength of concrete through the results of the non-destructive test. In this regard, simple linear regression with scatter plot is commonly adopted [9]. However, more advanced DoE, such as Response Surface [10], and even machine learning [11], have been successful in producing a more accurate prediction. For studies related to replacement of concrete materials [12,13], DoE approaches are highly favourable with a significant number of recent studies adopting more advanced methodology to analyse experimental data [14,15,16,17]. Various DoE has been used in the studies of replacement material such as tire rubber [14,18,19,20], palm oil fuel ash (POFA) [21,22,23], fly ash [22,24,25] and more. In another application, novel concrete mix design method, often with the incorporation of unconventional recycled material, is optimized through the use of curve fitting methods [26,27] or artificial intelligent based techniques [28,29].

While the account of its application varies between studies, certain methodologies are more commonly used than the others. Also, DoE is more frequently used in the prediction of concrete mechanical properties, but other applications, such as density, absorption and cost optimization are also available. Regression analysis is the simplest analytical method, which is employed to understand the relationship between variables. For experiments involving a large size of trials and data, the Taguchi method and Response Surface methodology are favoured to simplify the experimental process itself [30]. Another method is Artificial Neural Network which can be utilized for advanced analysis, particularly by researchers who are well-versed in the concept of computing. This paper presents a critical review on the usage, benefits, and challenges of the aforementioned DoE methods in the study of concrete materials.

## 2. Regression Analysis

### 2.1. The Concept of Regression Analysis

**Figure 1:**
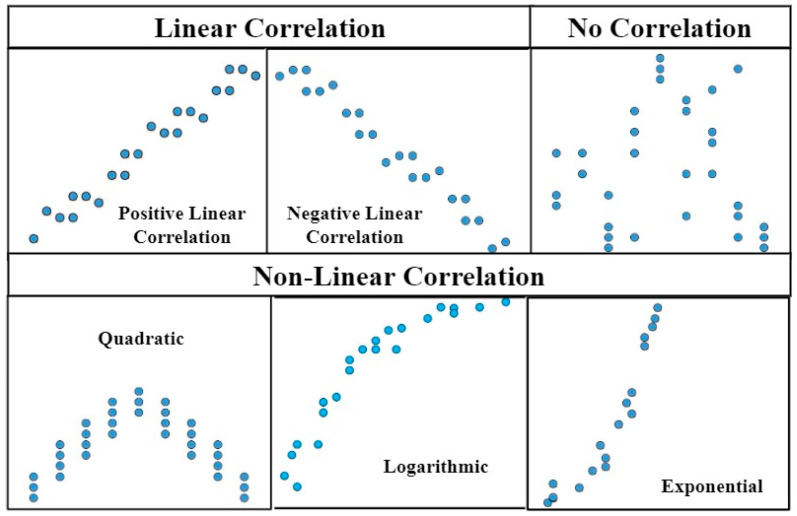
Scatterplot patterns [31].

Regression analysis is a basic statistical method that is still widely used to determine the relationship between a single dependent variable with other independent variables. A simple linear regression identifies and expresses two variables that are linearly related to each other. Using the most common tool such as Microsoft Excel or simple graph-plotting software, researchers can study the relationship between variables with little effort. By plotting two variables on graph, the relationship between them can be studied. Variables with linear relationship fall along a line, while non-linear relationship commonly depicts a curved pattern on the plot. If no pattern can be discerned, the variables are independent to each other. Figure 1 shows the general patterns of scatterplot.

While the most basic form of regression is used to test for linear relationship, the regression method is able to test for other relationships by transformation as shown in Table 1. A linear relationship is studied between a dependent variable (y) and independent variable (x) and then the relationship is transformed into second-order polynomial by running a multiple regression with the variable y and variables of x and x2. On the other hand, an exponential regression can be conducted with the same method by transforming the dependent variable into its logarithmic function, i.e., ln(y), and correlating it with the independent variable x. The ease and versatility of regression analysis explains its popularity among researchers in the engineering disciplines, including those with limited statistical knowledge. Apart from determining the relationship between two variables, the expression for a single dependent variable with many variables can be formulated using Multiple Linear Regression (MLR) or Mixed Regression, depending on whether all variables have a linear relationship with the dependent variable.

### 2.2. Applications of Regression Analysis

Different variations of regression analysis are widely used in the study of concrete materials. Regression analysis is used as a simple and accurate prediction for experiments similar to past studies, which indicated a linear relationship between variables. It is unclear whether polynomial expression may represent the data more accurately, but linear expression is considered sufficiently accurate for the analysis. Concrete studies that require a correlation expression, involving a single independent and dependent variable, largely utilize the regression analysis. For example, Ramana et al. [6] applied the regression method to evaluate the compressive strength of fiber reinforced concrete with 0 to 100% of recycled aggregate. The experiment returned an R^2^ value above 0.980 for all three conditions in the study, indicating that the regression model could be used for prediction with minimal deviation. A similar conclusion on non-destructive test was stated in the study by Kocáb, Misák, and Cikrle [32]; while a linear regression with high R^2^ does not invalidate more complicated expression, it can be used for achieving great effects within the scope of the study, and it is often sufficient. In another study [33], a similar regression with a single variable, namely concrete age, is used to predict the concrete strength under different curing conditions. The R^2^ value ranged from 0.87 to 0.98 for five cases, indicating that concrete age and compressive strength has strong linearity for various curing conditions. The expression allowed the researcher to study the effectiveness of each curing method by comparing the constant coefficient, as well as the slope, which indicates the rate of strength gain. However, it is worth noting that the graphical representation of the result showed the presence of curvature, which can potentially be represented more accurately by a polynomial regression. Hence, in order to consider applications of the method in the future, it is important to consider the sufficiency of a linear model, based on the scope of the study and the degree of potential accuracy improvement if a more complex model is to be used. While simple linear regression is commonly used to express the linear relationship between an independent variable and a dependent variable, the linear relationship between one variable with another can be expressed at a certain power, should the need arises. A modified regression method was performed by Halabe and Ray [34] for the purpose of verifying the theoretical relationship between compressive strength and ultrasonic pulse velocity to the power of four$\;(f_{ck\;}\alpha \;v^{4}$). While attempting to exclude an intercept, the researchers concluded that a linear relationship between the variables$\;(f_{ck\;}\alpha \;v$) resulted in a lower sum of square error (SSE) and higher R^2^. However, this does not imply that the method is not justified and should never be attempted to verify a theoretical relation.

On the other hand, multiple regression analysis has been utilized in concrete studies concerning more than one variable. The number of variables varies from as little as two [35] to as many as 10 [36]. Multiple linear regression (MLR) is the simplest form of multiple regression and is used in many research to obtain satisfactory results [35]. However, care must be taken as MLR only model the linear relationship between all the variables with the dependent variable while the inflence of certain variables may be non-linear. The effectiveness of MLR can be on par with other advanced methods such as Artificial Neural Network (ANN) in certain studies [37]. However, a more advanced statistical method would be more accurate for the modelling of analysis involving more variables [38]. While many studies use the classic MLR method, the backward method of the analysis is also viable as can be seen in the research of concreting productivity involving 10 factors [36]. To ensure the best MLR model, multicolinearity between predicting variables should be avoided. However, Aggarwal et al. [39] discovered that multicolinearity is present when using the proportion of concrete constietuents as the variables for concrete strength prediction, and used ridge regression to circumvent the problem. However, a practical application of a concrete strength prediction model has to make use of mix design proportion, and hence, most reviewed studies tend to disregard the effect of multicolinearity in the application of MLR. One method for compressing the information in the variables and eliminating multicolinearity is Principle Component Analysis (PCA). E. Garcia-Taengua [40] used PCA to combine three interrelated workability variables into an uncorrelated variable. In other cases where the interrelated variables are unknown [41,42], MLR was first applied normally before PCA was applied.

While performing a comprehensive analysis of different types of regression on the prediction of the strength of High Performance Concrete, Jin, Chen and Soboyejo [43] used the same data set, but conducted regression with the constituent of concrete expressed in kg/m^3^ versus ratio, in order to cement and percentages replacement. There is also no clear superiority between numeric variables method and relative method. MLR achieved a high accuracy of R^2^ = 0.907 despite not being the most accurate model. This is the overall trend observed in this review process. For very similar studies on the compressive concrete strength, MLR, logarithmic regression [44,45], and mix regression [4,43] were found to predict the dependent variable with great accuracy. On the other hand, exponential and second order polynomial regression are not favourable. The modified regression method with Fisher test has also been used to achieve a great effect in estimating the cost of concrete mixes [46]. Hence, researchers who intend to employ the regression analysis should utilize various types of regression analysis to obtain the most accurate expression. Based on the above reviews, it was also discovered that the common programs used for regression analysis are Minitab [43], SPSS [47], and MATLAB [35,38]. Table 2 summarizes the applications of the regression methods being reviewed.

## 3. Taguchi Method

### 3.1. The Concept of the Taguchi Method

The Taguchi method is a modified DoE method invented by a Japanese scientist named Dr Genechi Taguchi in the 1940s, about 25 years after the introduction of the original DoE by R.A. Fisher [50]. The original DoE method requires either the full factorial method of conducting experiment on all $n^{r}$ number of combinations or determinations of the optimal condition for every variable by testing one variable at a time (OVAT). This is especially true for many experiments, which utilize the classic regression or the MLR method. To reduce the number of tests required, the fractional factorial method was developed. The Taguchi method aims to minimize the number of testing using its own method called the Orthogonal Array. The Orthogonal Array is presented as preset tables, whereby details of the number of experiments, as shown in Table 3, are required to predict the dependent variable, based on the number of variables and values each variable can take. While the underlying principle of the Orthogonal Array is complex, the method can be used by simply following the preset tables once a basic understanding of the method is attained [51]. Table 4 shows the commonly used arrays for the experiment design. As indicated in the table, the Taguchi Orthogonal Array is extremely effective at minimizing the number of trials needed for the experiments and also capable of cutting down complex experiments with over a million numbers of full factorial combinations into only 32 trials. Taguchi also requires less trials without overlooking interaction between variables which is a major weakness of the OVAT method [52]. Such appeal earns it many usage in the research of concrete materials which is cost, time, and labor intensive [53].

### 3.2. Applications of Taguchi Method

In most studies that applyi the Taguchi method, researchers are concerned with determining the quantity or proportion of each constituent material that is needed to produce a concrete mix design with the best strength and performance. While this method showed promising results in all of the reviewed literatures, only two studies [54,55] attempted to develop an expression for predicting the results. Specifically, Shiri et al. [54] conducted ANOVA on top of the Taguchi analysis to obtain the significance of each variable based on 95% confidence level and developed an accurate expression using the regression analysis. In similar vein, Abbasi et al. [55] who conducted the regression analysis using the data set similarly advised by the Taguchi Orthogonal Array also obtained an accurate expression for the relationship between variables and the compressive strength and electric resistance of concrete. However, the expression for permeability is less accurate, with R^2^ value of 0.634. With that in mind, this paper reviewed the existing literature and conducted the regression analysis on the data set from each study to verify this phenomena. In each reviewed paper, the experimental regime was duplicated, and the data were manually filled in from the Taguchi Orthogonal Array if it was not presented in the studies. Studies which did not include the parameters in detail were neglected [56]. MLR was firstly conducted on the data sets. When the result was not satisfactory (R^2^ < 0.80), the main effect plot from Taguchi analysis was referred to and any non-linear effect of the variable was transformed into an appropriate function. Then, the mixed regression analysis was conducted. From the output, any variable which was not significant was excluded. Finally, the regression analysis was carried out for the remaining variables and the final R^2^ was reported, as shown in Table 5.

The table above shows that the regression analysis on orthogonal array produced satisfactory result for most studies. Porosity [53], compressive strength [59,60,62], flexural strength [58], and even water absorption [60] of concrete can be accurately expressed using this method. The concrete compressive strength at seven days can be accurately estimated in the experiment by Hadi et al. [61]. However, the derived expression failed to predict the compressive strength of self-compacting concrete at 14 days in another experiment by Teimortashlu, Dehestani, and Jalal [59]. Therefore, more studies may be required to explain this deviation. Some plausible reasons could be due to the selection of variables and the behavior of self-compacting concrete. The regression method also failed in the study byArulraj et al. [27] whereby three of the five variables only have two levels, resulting in insufficient data, in order to develop an accurate expression. Despite certain shortcomings, the method is generally successful in providing information for a more detailed experimental analysis. It is recommended that future applications of the Taguchi method may utilize the regression analysis on Orthogonal Array.

The tables provided for the Taguchi Orthogonal Array were also found to be helpful as most of the reviewed studies have adopted one of the preset arrays without the need for additional technical modification. L-9 matric is useful for small-scale material studies, while modified L-16 matric is suitable for mix design optimization due to the large number of variables influencing the concrete performance. For the studies that require a matric outside of the provided presets, the development of a unique matric, fitting a different number of factor and level is possible. Abbasi et al. [55] modified the preset L-18 matric into a unique matric with 12 levels. In another event, a larger matric can be used in experiments with fewer factors by assuming an empty column on the selected array. For example, L-9 array, which accommodates four level-three factors [56,57] can be used in experiments with only three level-three factors [60,61]. The above findings suggest that the Taguchi method is a highly optimized method to deliver accuracy results in concrete-related experiments.

## 4. Response Surface Methodology (RSM)

### 4.1. The Concept of the RSM

The RSM is another DoE method which evaluates the effect and interaction of multiple variables on a dependent variable. Just like the Taguchi method, the primary purpose is to simplify the experimental process and optimize the responses. According to Bradley [62], the RSM mechanism involves understanding the topography of the response surface, including the local maximum, local, minimum and ridge lines and also find the region where the most appropriate response occurs. As shown in Table 6, the RSM considers the first order, second order, and interaction effects between the variables, in order to formulate a response surface that determines the optimum condition for the dependent variable. Like all DoE methods, the RSM provides a mathematical solution to a problem, reduces the number of experimental trials, and saves the cost and time in the study conducted [63]. It also includes the interaction effect of variables to improve the accuracy of the model. However, one disadvantage of the RSM is that the experimental data are fitted to a second-order polynomial order, even though it may not be the most suitable model for expressing all systems with curvature [64].

Although the RSM offers a sophisticated analysis tool for experimental data, it does not specify the methodology by which data should be collected. Unlike the Taguchi method, which adheres to the Orthogonal Array, multiple methodologies exist for the data collection process. The methods include Box-Behnken Design (BBD), Central Composite Design (CCD), Doehlert Matrix (DM), three-level full factorial designs, and others. Three-level factorial design involves conducting experiments on all possible combinations, and hence, has limited applications in relation the RSM, as the number of experiments required becomes too large when the number of factors increases, causing low efficiency in data collection [65]. The efficiency of the other three methods were also being studied from other studies [66,67], and the summary of findings is tabulated on Table 7. From the table, DM was found to be the most efficient model for data selection. One advantage of the DM method is that it uses different number of levels for the variables, allowing a variable with known stronger effect to be assigned with more levels for detailed analysis [66]. The second most efficient method is the BBD. The BBD method can be considered for any experiment that may result in inaccuracy if it is performed at the extreme conditions, since such experiment does not contain combinations for which all factors are at their highest or lowest levels [67]. This benefit is especially relevant, and thus, should be taken into consideration for the studies of concrete materials.

### 4.2. Applications of the RSM

Table 8 summarizes the information obtained from the relevant literatures reviews. Reviews on the efficiency of data collection methods [66,67] indicates that the DM and BBD methods are the most efficient, but most of concrete-related studies used the CCD method in designing the experiments. The reason for selecting this method was not specified in several studies [68,69,70]. Yet, according to Nambiar and Ramamurthy [71], the CCD allows equal precision of estimates in all directions. Meanwhile, other researchers [72,73] used CCD due to its rotatability and ability to predict the result within experimental range with great precision. Of all the literature reviewed, many researchers used the Minitab software to conduct the RSM experimental design and data analysis [74,75,76]. Design Expert were also favored by several others [68,69], while one study used the Statistical Analysis System (SAS) to perform the experiment [71]. Since BBD and CCD are the two methods provided in Minitab, this may explain why the said methods are frequently adopted. While the RSM is able to handle larger number of variables, most researchers use it to design experiments involving two [75,77,78,79] or three [68,69,70] variables, presumably to make full use of the contour plot, which can represent the effect of two variables as commonly used in design codes such as Eurocode 2 [70].

In certain studies, workability of concrete, as indicated by slump test result, was selected as an dependant variable of RSM analysis. While the workability of concrete can be measured at the early phase of concrete production, the inclusion of slump value is mainly done to study the inflence of replacement material on concrete workability through the RSM model. For example, in the study of concrete with electronic waste [68] and rubber waste [80], the workability of concrete with respect to water-cement ratio was studied through RSM contour plot. On the other hand, Nambiar and Ramamurthy [71] forumulated a prediction model for foam concrete workability for the development of mix design. Similarly, Şimşek et al. [72] used surface plot to optimize the mix proportion of concrete. Apart from producing the response surface to study the optimal condition of independent variables that gives the highest performance, researchers frequently perform further analysis to obtain the equation for the prediction of the dependent variable. One method is by conducting ANOVA on the results [74,75,81] to determine the significance of each term using Student’s t-test. In this process, terms which are deemed insignificant are removed. While most studies use the convention rule of taking a confidence level of 95% with the threshold of *p*-value < 0.05 to determine the significance of each variable in the analysis, researchers have attempted to adopt lower confident levels to include more variables in the final model. For example, Mrudul et al. [73] used 90% confidence level (*p*-value <0.10) in t-test, but the final model remained satisfactory with R^2^ value above 0.90, which indicate that 90% variation in compressive strength of silica infused recycled aggregate concrete can be attributed to the variables. Meanwhile, Vasudevan, Poornima, and Balachandran [76] optimized the output with 85% confident level. The R^2^ value of the original RSM model was 0.980, but after dispensing terms with *p*-value above 0.15, the R^2^ became 0.975, which was a negligible drop even though the process eliminated 3 terms from the RSM model. This hints that the standard convention of 95% confidence interval does not need to be absolutely followed when optimizing equations. The ability of the RSM in predicting the concrete properties is satisfactory. Even though the RSM only presents data in the second-order polynomial or quadratic form [64], reviews of existing studies showed that this does not impact the accuracy of the model. The inclusion of the interaction effect improves the model significantly. The Response Surface Regression provides a detailed analysis and accurate estimation of mechanical properties [14]. In addition, other properties such as permeability, sorptivity [76] and water absorption [14] of concrete can be modelled or predicted.

## 5. Artificial Neural Networks (ANNs)

### 5.1. The Concept of ANNs

**Figure 2:**
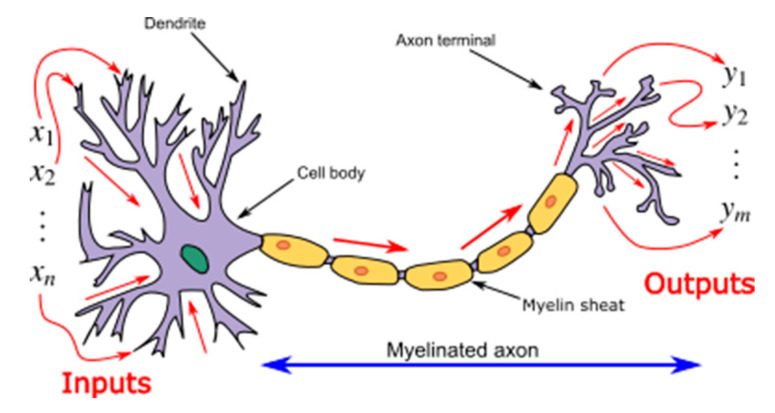
Function of neurons in human thinking process [79].

**Figure 3:**
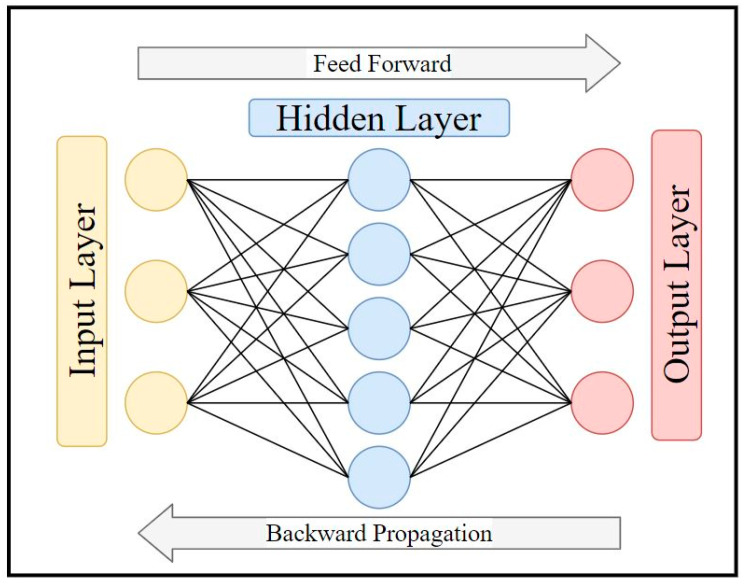
Basic schematic of ANNs [81].

The Neural Network is a web of interconnected neurons, which conduct parallel processing during the thinking process, and where millions of neurons transmit signals to each other to process information [79]. In the human brain, neurons receive sensory input from the external world via dendrites, process it and give the output through axons, as shown in Figure 2. ANNs are an advanced analysis methodology, which simulate the thinking process of the human brain [81]. Mathematically, ANNs are used to process a number of inputs and provide an output, similar to other DoE methods, which take in multiple variables to predict the dependent variable. Figure 3 shows the basic schematic of the ANNs. The mechanism of ANNs involves three layers, which are the input layer, hidden layer, and output layer. The input layer is where data are inserted. A system of weighted connections is used to process the data and return the result at the output layer. The process begins with a feed-forward of the inputs and ends with the output. Then, the weight of connections needs to be optimized, usually by backward propagation. The difference between the predicted value and actual value is considered to adjust and modify the mechanism of the hidden layer. ANNs have a series of advantages and disadvantages. For data analysis, the most pronounced advantages are the ability to tolerate error in the system due to their processing [82], and the ability to solve complex non-linear relationship between variables [83]. The resistance to a faulty system also extends their ability to work with incomplete data [84]. ANNs are advantageous compared to programmed computer algorithms as they can improve their own rules through the number of decisions made [85]. On the other hand, the solution provided by ANNs is often not described [79], and its complexity can be prone to overfitting of data.

### 5.2. The Applications of ANNs

Table 9 summarizes the information obtained from the reviews of ANNs-related literature. As observed, a variety of tools and software are used by researchers to conduct ANNs. MATLAB, with the Neural Network Fitting Tool [86], remains a popular choice that has been used in many studies [38,87,88]. Other than that, several researchers used other software such as JMP [74], QBasic [89], Neuro Solutions [90], and WEKA [91]. Unlike other DoE methods reviewed, ANNs are a more complex system which requires various setup steps. First, the perceptron needs to be constructed by setting up the number of input nodes, hidden layer, and hidden nodes. The number of input nodes is simply the number of variables in the studies. However, there is no requirement for a fixed number of hidden layer and nodes. In several concrete-related studies, the researchers only used one or at most two hidden layers [89,92]. However, the number of neurons in the hidden layer largely varies by studies. The more hidden layer neurons are introduced into the perceptron, the more memorizing power and the less reasoning capability the system holds [93]. Hence, the number of neurons should be kept minimal but enough to simulate the training data. A rule of thumb for the maximum number of neurons is $N_{H}\leq \;2N_{i}+1\;$where $N_{H}$ represents the number of neurons and $N_{i}$ represents the number of inputs. This convention was referred to by some researchers [38,90] when deciding the number of neurons. However, most studies performed trial-and-error to obtain the most suitable model for each respective experiment [93], and not all studies adhere to the rule of thumb [94].

Another important consideration of ANNs is to specify the amount or portion of data on training, validation, and testing of the perceptron. Algorithm training is important, and incorrect or insufficient training will result in poor quality of the model. Several researchers applied the training algorithm rather than the manual selection. K-fold cross validation [93,95] and Levenberg-Marquardt training algorithm [86,88] provided by MATLAB are commonly used. If it is decided that no algorithm will be used, the researchers devote a portion of the available data set for training, validation, and testing process. A huge majority of the data is allocated for the system training, ranging from 50% to 80%. The remaining data is usually distributed evenly for validation and testing [87,90,92]. In certain studies, only the training-to-testing ratio was given [16,68], but the proportion skewed heavily towards training. Khashman and Akpinar [98] who conducted the same ANNs models with a training-to-testing ratio of 40–60, 50–50, and 60–40 concluded that model with a ratio of 50–50 is the best. However, this should not be used as the absolute guideline as the experiment had a huge amount of data (i.e., 1030). More studies are still needed to formulate conclusions on this decision, and researchers are advised to experiment with various proportions for achieving the best result. Another setting required for back-propagation ANNs is the training rate, momentum, and iteration, which is summarized in Table 10.

ANNs are widely used to predict the concrete compressive strength with number of variables more than the commonly used for other DoE methods. One unique application of ANNs is that the advanced computing power of the method allows the meta-analysis of several concrete studies that use the same replacement materials. Gupta [95] who collected 32 data from 10 different literature on concrete containing nano-silica formulated an accurate model for 28 days concrete compressive strength without the need to perform any experiment. In another study, Asteris and Mokos [88] used 209 data sets from a thesis [99] and performed ANNs on the prediction of concrete strength using the non-destructive tests result. A similar analysis was conducted by Noorzaei et al. [89] and Santosa and Purbo Santosa [97] who also achieved the same success using the constituents of concrete as variables. The accuracy of ANNs, as denoted by the R^2^ value, is superior to regression analysis [38,90], including multiple non-linear regression [92]. However, in one study on self-compacting concrete [37], the result of MLR provided a higher R^2^ value than the model produced from ANNs. This may be attribute to the low number of data in the experiment (i.e., 15), as ANNs perform better when more data is being fed. In addition, the R^2^ value should not be the sole factor used to decide on the best model. In another experiment on recycled aggregate concrete [74], both RSM and ANNs methods provided high R^2^ values but the Root Mean Squared Error (RMSE) of the ANNs model was significantly lower than the other models.

To model the concrete compressive strength at a certain age, most studies tend to include the constituents of concrete as the variables, and a model is produced for every date concerned. However, an alternative methodology was conducted by Chopra et al. [94], whereby in their experiment, six variables of concrete constituent were used to predict the 28-day concrete strength. As for 56-day concrete strength, the 28-day strength was added as an additional factor, and the 56-day strength was added again for the prediction of 90-day strength in the study. This method allows the strength gain to be studied in more detailed. Meanwhile, Atici [87] used the regression model to create six models of different combinations of significant variables before applying ANNs to determine the best solution. In another study, the ANNs method was compared with Genetic Programming [94], but the differences in accuracy are indiscernible as both methods produce highly accurate models. However, Chandwani et al. [100] proposed the hybridization of ANN and Genetic Algorithm (GA), which improved the convergence speed and accuracy of the model [101] and helped in the derivation of optimal result [102]. ANN-GA is currently not too widely applied in concrete material studies, but has seen usage in complex studies involving more advanced technologies, such as self-healing concrete [103]. Overall, the literature reviews indicate that ANNs is a complex, but powerful DoE method that allows researchers to perform the advanced analysis of concrete performance.

## 6. Conclusions

This paper discussed the concept and applications of the DoE methods in the research regarding concrete mechanical properties. In the field of concrete materials, the concrete performance is affected by a multitude of variables, which makes it impractical to study and experiment an innovation through sheer experimentation. DoE offers a solution for minimizing the number of experiments to conserve time, money and labor, as well as provide a superior data analysis methodology that can give accurate results and predictions. When applying DoE, and especially in complex analysis, it is important to ensure the physical meaning of the variables is sound, instead of merely seeking the strongest correlations, as mathematical correlation does not necessary imply logical causation. The mixed regression analysis is a versatile technique that can provide an expression for the properties of sustainable concrete, through either single or multiple variables. The combination of linear and logarithmic functions is widely used in the modelling of concrete properties. Regression analysis has adequate accuracy, but it is less accurate compared to other advanced techniques. The Taguchi method is applied to minimize the number of experiments required to a huge extent using the Orthogonal Array. It is effective and easy to use, provided that the number and level of variables fit into the array. L-9 matrix is suitable for the study on replacement of concrete materials while modified L-16 matrix is largely used for design mix optimization. The RSM is mainly used in concrete material studies with two or three variables to produce response surface which is similar to information exist in the design standard. CCD is a commonly used method in organizing the data for the experiment. Last, ANN is an advanced method of analysis that requires the use of cross-discipline knowledge in concrete-related studies, but it can provide a highly accurate expression when a sufficiently large amount of data is available. In studies concerning sustainable concrete, DoE methods have shown successful results concrete properties, the effect of replacement materials, and the development of concrete mix design. However, mechanical properties of concrete are currently the dominant application of DoE with application on other properties to be explored.

## Figures and Tables

**Table 1:** Regression expression for different relationships.

Types of Regression	Expression	Dependent Variable	Independent Variable
Linear	y=mx+c	y	x
Second-order polynomial	$y=Ax^{2}+Bx+C$	y	$x,\;x^{2}$
Exponential	$y=Ae^{x}$	ln(y)	x
Logarithmic	$y=Ax^{b}$	ln(y)	ln(x)
Types of Regression	Combination of the above		

**Table 2:** Application of regression analysis.

Sources	Dependent Variable	No. of Variables	Methodology	R^2^
[6]	Compressive strength	1	Linear Regression	0.890
[32]	Compressive strength	1	Linear Regression	0.878–0.963
[33]	Compressive strength	1	Linear Regression	0.870–0.980
[34]	Compressive strength	1	Modified LR	–
[35]	Compressive strength	1	Modified LR	–
[35]	Stress-strength ratio	2	Classic MLR	0.738
[48]	Compressive strength	5	Classic MLR	0.82
	Slump	5	Classic MLR	0.73–0.88
[49]	Compressive strength	5	Classic MLR	0.612
[37]	Compressive strength	4	Classic MLR	0.962
[47]	Compressive strength	7	Classic MLR	0.96–0.98
[38]	Compressive strength	8	Classic MLR	0.800
[44]	Compressive strength	6	Logarithmic Regression	0.758–0.866
[45]	Compressive strength	8	Logarithmic Regression	0.999
[36]	Compressive strength	10	Backward MLR	0.857
[46]	Concreting productivity	4	Modified Regression	–
[43]	Cost of concrete	9 (numeric)	Classic MLR	0.907
	Compressive strength	8 (relative)	Exponential Regression	0.876
	Compressive strength	8 (relative)	Logarithmic Regression	0.953
	Compressive strength	8 (relative)	Mixed Regression	0.740–0.914
[4]	Compressive strength	8	Mixed Regression	0.844

**Table 3:** L-4 Orthogonal Array.

Trail	Factors		
	A	B	C
1	1	1	1
2	1	2	2
3	2	1	2
4	2	2	1

**Table 4:** Taguchi orthogonal array.

Array	Factors	Full Factorial Combinations	OVAT	Taguchi
L-4	3 two-level factors	8	6	4
L-8	7 two-level factors	128	14	8
L-12	11 two-level factors	2048	22	12
L-16	15 two-level factors	32,768	30	16
L-32	31 two-level factors	2,147,483,648	62	32
L-9	4 three-level factors	81	12	9
L-18	1 two-level and 7 three-level factors	4374	23	18
L-27	13 three-level factors	1,594,323	39	27
L-16 *	5 four-level factors	1024	20	16
L-32 *	1 two-level and 9 four level factors	524,288	38	32

* Modified array.

**Table 5:** Taguchi method and regression analysis.

Sources	Dependent Variable	Factors	Level	Array	R^2^
[53]	Porosity	4	4	L-16 *	0.813
[57]	Compressive strength	4	3	L-9	0.963
[56]	Compressive strength	4	3	L-9	-
[58]	Dry density	5	4	L-16 *	0.637
	Flexural strength	5	4	L-16 *	0.900
[59]	14d Compressive strength	3	4	L-16 *	0.528
	28d Compressive strength	3	4	L-16 *	0.720
[60]	Compressive strength	3	3	L-9	0.954
	Water absorption	3	3	L-9	0.960
[54]	Compressive strength	4	3	L-9	0.962
[55]	Compressive strength	3	2, 3, 12	L-18 *	0.911
	Electric resistance	3	2, 3, 12	L-18 *	0.801
	Permeability	3	2, 3, 12	L-18 *	0.634
[27]	Compressive strength	5	2, 4	L-16 *	0.374
	Tensile strength	5	2, 4	L-16 *	0.449
[61]	7d Compressive strength	3	3	L-9	0.905
	28d Compressive strength	3	3	L-9	0.890

* Modified array.

**Table 6:** General expression of RSM for two independent variables.

Effect	Term
Intercept/Constant	$B_{0}$
First order	$B_{1}x_{1}$, $B_{2}x_{2}$
Second order	$B_{11}x_{1}^{2}$, $B_{22}x_{2}^{2}$
Interaction	$B_{12}x_{1}x_{2}$
General Expression	$y=B_{0}+B_{1}x_{1}+B_{2}x_{2}+B_{11}x_{1}^{2}+B_{22}x_{2}^{2}+B_{12}x_{1}x_{2}$

**Table 7:** Efficiency of various DoE methods.

Variables (K)	Number of Coefficient (*p*)	Number of Experiments (f)			Efficiency (*p*/f)		
		CCD	DM	BBD	CCD	DM	BBD
2	6	9	7	-	0.67	0.86	-
3	10	15	13	13	0.67	0.77	0.77
4	15	25	21	25	0.60	0.71	0.60
5	21	43	31	41	0.49	0.68	0.61
6	28	77	43	61	0.36	0.65	0.46
7	36	143	57	85	0.25	0.63	0.42
8	45	273	73	113	0.16	0.62	0.40

**Table 8:** Summary of the RSM studies.

Sources	Dependent Variable	Factor	Method
[77]	Compressive strength	5	BBD
[75]	Compressive strength	2	-
[74]	Compressive strength	3	CCD
[78]	Cement-SP compatibility	3	CCD
[71]	Compressive strength, dry density	3	CCD
[72]	Slump, compressive strength, split tensile strength	3	CCD
[68]	Slump, density, compressive strength, split tensile strength	2	CCD
[73]	Compressive strength	2	CCD
[76]	Compressive strength, permeability, sorptivity	3	CCD
[69]	Mechanical properties	2	CCD
[14]	Mechanical properties, water absorption	3	CCD

**Table 9:** Summary of ANNs studies.

Sources	No. of Variables	No. of Hidden Nodes	No. of Data	Training-to-Testing Ratio	R^2^
[38]	7	15	140	85-15	0.961
[37]	4	NS	15	NS	0.898
[74]	4	4	17	NS	0.980
[87]	3-5	4–6	28	70–15–15	0.891–0.990
[89]	6	12,6	639	63–15–22	-
[95]	7	4	32	k-fold	0.869
[90]	7	4	173	80–10–10	0.899
[92]	5	6, 6	2340	60–20–20	0.999
[96]	3	3	12	NS	0.970
[86]	8	NS	1030	Levenberg-Marquardt algorithm	0.916
[93]	6, 8	8	80, 31	k-fold	0.919–0.969
[97]	7	8	103	NS	NS
[91]	5	NS	55	NS	0.879–0.893
[94]	4–6	50	49, 27	75–25	0.898–1.000
[98]	9	NS	1030	50–50	0.860
[88]	2	NS	209	Levenberg-Marquardt algorithm	0.800–1.00

**Table 10:** Summary on the settings of back-propagation ANNs.

Sources	Training Rate	Momentum	Iteration
[37]	0.04	0.1	500
[89]	0.08	0.65	10,000
[93]	0.04	0.1	500
	0.6	0.3	500
	0.5	0.2	1000
	0.2	0.1	1300
[91]	0.2	0.1	400

## Data Availability

The data presented in this study are available on request from the corresponding author.
